# Detection of *Plasmodium falciparum* infected *Anopheles gambiae* using near-infrared spectroscopy

**DOI:** 10.1186/s12936-019-2719-9

**Published:** 2019-03-19

**Authors:** Marta F. Maia, Melissa Kapulu, Michelle Muthui, Martin G. Wagah, Heather M. Ferguson, Floyd E. Dowell, Francesco Baldini, Lisa-Ranford Cartwright

**Affiliations:** 1 Swiss Tropical and Public Health Institute, Socinstrasse 57, 4020 Basel, Switzerland; 2 University of Basel, Petersplatz 1, 4001 Basel, Switzerland; 3 KEMRI Wellcome Trust Research Programme, P.O. Box 230, Kilifi, 80108 Kenya; 4 Centre for Tropical Medicine and Global Health, Nuffield Department of Medicine, University of Oxford, Old Road Campus Roosevelt Drive, Oxford, OX3 7FZ UK; 5 Department of Public Health, School of Human and Health Sciences, Pwani University, Kilifi, Kenya; 6 Institute of Biodiversity, Animal Health and Comparative Medicine, University of Glasgow, Graham Kerr Building, Glasgow, G12 8QQ UK; 7 USDA, Agricultural Research Service, Center for Grain and Animal Health Research, 1515 College Avenue, Manhattan, KS 66502 USA

**Keywords:** Near infrared spectroscopy, Malaria, *Anopheles gambiae*, *Plasmodium falciparum*, Sporozoite, Oocyst, Partial least square regression, Africa, Vector

## Abstract

**Background:**

Large-scale surveillance of mosquito populations is crucial to assess the intensity of vector-borne disease transmission and the impact of control interventions. However, there is a lack of accurate, cost-effective and high-throughput tools for mass-screening of vectors.

**Methods:**

A total of 750 *Anopheles gambiae* (Keele strain) mosquitoes were fed *Plasmodium falciparum* NF54 gametocytes through standard membrane feeding assay (SMFA) and afterwards maintained in insectary conditions to allow for oocyst (8 days) and sporozoite development (14 days). Thereupon, each mosquito was scanned using near infra-red spectroscopy (NIRS) and processed by quantitative polymerase chain reaction (qPCR) to determine the presence of infection and infection load. The spectra collected were randomly assigned to either a training dataset, used to develop calibrations for predicting oocyst- or sporozoite-infection through partial least square regressions (PLS); or to a test dataset, used for validating the calibration’s prediction accuracy.

**Results:**

NIRS detected oocyst- and sporozoite-stage *P. falciparum* infections with 88% and 95% accuracy, respectively. This study demonstrates proof-of-concept that NIRS is capable of rapidly identifying laboratory strains of human malaria infection in African mosquito vectors.

**Conclusions:**

Accurate, low-cost, reagent-free screening of mosquito populations enabled by NIRS could revolutionize surveillance and elimination strategies for the most important human malaria parasite in its primary African vector species. Further research is needed to evaluate how the method performs in the field following adjustments in the training datasets to include data from wild-caught infected and uninfected mosquitoes.

**Electronic supplementary material:**

The online version of this article (10.1186/s12936-019-2719-9) contains supplementary material, which is available to authorized users.

## Background

Malaria is holding back development in endemic countries and remains one of the leading causes of death in children under 5 years-old in sub-Saharan Africa [1–3]. During the past decade, the large-scale roll-out of long-lasting insecticide-treated nets and indoor residual spraying across Africa has resulted in a substantial reduction in malaria cases [4]. The Global Technical Strategy for Malaria 2016–2030 of the World Health Organization (WHO) seeks to reduce malaria incidence and related mortality by at least 90% and to eliminate the disease in a minimum of 35 countries [1]. These bold goals will require new interventions that can address residual malaria transmission as well as new tools to better monitor their impact on vector-borne disease transmission. Mosquito surveillance is a cornerstone of the control of malaria and other vector-borne diseases [5].

However, presently, there is no high-throughput, cost-efficient method to identify *Plasmodium* infection and infectiousness in mosquitoes. Molecular methods such as ELISA and PCR are used to determine parasite infection, but these are expensive and laborious [6–8], challenging resource-poor countries with few funds and limited access to reagents and equipment, and thus are unsuitable for large-scale surveillance. A further complication is that typically only 1–2% of mosquitoes may be infected with transmission stage parasites (sporozoites), meaning that very large sample sizes must be tested to accurately quantify site and time-specific estimates of mosquito infection rates as will be required to assess progress towards malaria elimination [9].

Recent advances indicate several mosquito traits can be accurately identified through analysis of their tissues with near infrared spectroscopy (NIRS) [10–13]. This method involves the passing of visible and NIR light (wavelength 400–2500 nanometres) through the whole or part of a mosquito specimen and the collection of an absorbance spectrum instantly, without destroying the sample. Changes in spectral peaks at different wavelengths represent how intensely different molecules absorb light, and thus NIR spectra of mosquitoes are determined by the biochemical composition of their tissues, which are known to differ according to age [14, 15], species [16, 17], microbiome [18], physiological stage [19, 20], and pathogen infection status [20, 21]. Differences in NIR spectra have been used to distinguish young (e.g. < 7 days old) from older (7 + days old) malaria vectors, to identify morphologically identical *Anopheles* sibling species, and to detect the presence of the endosymbiont *Wolbachia* in *Aedes aegypti* mosquitoes [10–12]. Most recently, NIRS has been used to detect rodent malaria infections in laboratory-reared *Anopheles stephensi* [22] and Zika virus in *Aedes aegypti* [23]. The use of NIRS has not previously been investigated on human malaria infected mosquitoes. The presence of the parasite-specific proteins and other biochemical changes induced by malaria infection in the vector may permit these to be distinguished from uninfected mosquitoes using spectral tools such as NIRS [24, 25].

Parasite infection in the mosquito can be found in two main forms defined by their parasite development stages: midgut oocyst infections occurring around 2–8 days after feeding on infectious blood; and sporozoite infections occurring 9–14 days after infection, characterized by the release of sporozoites from oocysts into the mosquito’s haemocoel and salivary glands, enabling the mosquito to infect the next human host. Given the different nature of the two infection stages the NIRS profile of an oocyst-infected mosquito may not be the same as a sporozoite-infected one. For this reason, this study aimed to test whether NIRS could successfully identify oocyst and sporozoite infections in *Anopheles* vectors, and estimate if the method’s prediction accuracy is dependent on the intensity of infection in the mosquito.

This paper presents the successful application of NIRS to differentiate *Plasmodium falciparum*-infected mosquitoes from uninfected mosquitoes, providing the first evidence of detection of human malaria infections in the *An. gambiae* mosquito vector by this cost-effective, fast and reagent-free method. The development of a tool such as NIRS to measure malaria infection rates in mosquito populations would be of great service to malaria pre-elimination efforts as it would allow the processing of large numbers of mosquitoes increasing the accuracy of the estimates of human exposure to malaria infection across different regions, and advancing malaria vector surveillance in Africa.

## Methods

### Mosquitoes

Mosquitoes from a colony of *An. gambiae* sensu stricto (Keele line) [26] were reared under standard insectary conditions (26 ± 1 °C, 80% humidity, 12 h light:12 h dark cycle) at the University of Glasgow, Scotland, UK. Larvae were fed on Tetramin tropical flakes and Tetra Pond Pellets (Tetra Ltd, UK). Pupae were transferred into cages for adult emergence. Adult mosquitoes were fed ad libitum on 5% glucose solution containing 0.05% (w/v) 4-aminobenzoic acid (PABA). SMFA was done with 3–6 days old mosquitoes.

### Parasite culture and standard membrane feeding assays (SMFA)

*Plasmodium falciparum* (NF54) parasites were cultured using standard methodology to produce infectious gametocytes [27], using human blood and serum obtained from the Glasgow and West of Scotland Blood Transfusion Service. Standard membrane feeding assays (SMFA) were conducted on three different occasions using gametocytes produced in vitro: the first SMFA was done with a high gametocyte density (approx. 1% gametocytes) and the two-subsequent feeds with a lower density (~ 0.1% gametocytes) to produce more uninfected mosquitoes. For each SMFA, 300 female *An. gambiae* (Keele line) 3–6 days post emergence were distributed in pairs into 6 cups of 50 mosquitoes each. In the first SMFA, mosquitoes were 3, 4 and 5 days old, in the second SMFA they were 4, 5 and 6 days old and in the third SMFA mosquitoes were 3 (2 pairs of cups) and 4 days old. One cup of each pair was offered blood with infectious gametocytes and allowed to feed for 20 min. The temperature of the membrane feeders was then reduced to below 30 °C for 30 min to allow all mature gametocytes to complete gametogenesis [28]. The remaining cups of mosquitoes were then allowed to feed on the same blood, to produce control mosquitoes with zero infection rates, and thus obtain a comparable control sample differing only in the complete absence of parasite infection.

### Near infrared spectra collection and data analysis

After feeding, the blood-fed mosquitoes in each pot were maintained for 14 days under insectary conditions and examined for oocyst and sporozoite development on day 7 and 14 days post-infection, respectively. Mosquitoes were killed using chloroform vapour before collecting near infrared absorbance spectra from each individual mosquito without any further processing, using a Labspec 4i NIR spectrometer with an internal 18.6 W light source (ASD Inc, Longmont, CO) and ASD software RS^3^ per established protocols [10], but using a 3.2 mm-diameter bifurcated fibre-optic probe which contained a single 600 micron collection fibre surrounded by six 600 micron illumination fibres. The probe was placed 2.4 mm from a spectralon plate onto which the mosquitoes were placed for scanning. All mosquitoes were scanned on their cephalothorax. Spectra between 500 and 2400 nm were analysed through leave-one-out cross validations (LOOCV) using partial least square (PLS) regression in GRAMS Plus/IQ software (Thermo Galactic, Salem, NH). After scanning, each mosquito carcass was stored individually at − 80 °C in ATL lysis buffer (QIAGEN) until DNA extraction, to perform qPCR to determine the infection status of the mosquito.

### DNA extraction and quantitative real-time polymerase chain reaction (qPCR)

DNA was extracted using Qiagen DNeasy Blood & Tissue^®^ DNA extraction kits from mosquito abdomens (for mosquitoes analysed 7 days post infectious feed) and whole mosquitoes (for mosquitoes killed 14 days post infectious feed) and eluted in 50 µL of water. A 20 µL aliquot of the 50 µL of extracted DNA for each mosquito was transferred to individual wells of DNAstable^®^ 96 well plates (Sigma-Aldrich) and allowed to air dry at room temperature. The plates were shipped to KEMRI Wellcome Trust (Killifi, Kenya) for qPCR analysis. Samples were reconstituted in 20 µL of DNAse-free water and *P. falciparum* genome numbers present were quantified by qPCR [29]. Quantification reactions were performed in 15 μL volumes, containing 1.2 μL of 10 mM forward and reverse primers (377F: 5′ ACTCCAGAAGAAGAAGAGCAAGC-3′; 377R: 5′-TTCATCAGTAAAAAAAGAATCGTCATC-3′); 7.5 μL of SYBR^®^ Green PCR Master Mix, 1.1 μL of DNAse-free water and 4 μL of sample DNA, using an Applied Biosystems 7500 Real-Time PCR System. The cycling profile comprised an initial denaturation of 95 °C for 900 s (holding stage) and then 40 amplification cycles of denaturation 95 °C for 30 s (seconds), annealing 55 °C for 20 s and extension 68 °C for 30 s. At the end of amplification, melt curves were produced with 15 s denaturation at 95 °C, followed by 60 s at 60 °C, 30 s at 95 °C and 15 s at 60 °C. Parasite load was estimated for each sample by comparison with the standard curve drawn from the DNA standards using Applied Biosystems 7500 software v2.0.6. Samples which amplified after 38 cycles, or which showed a shift in melt curve or two melt curve peaks were excluded.

DNA extracted from uninfected mosquitoes (abdomens and cephalothorax) were used as negative controls, in addition to negative controls with no DNA. Standard curves were generated for each qPCR run using a 5-point tenfold serial dilution of DNA extracted from asexual 3D7 cultures synchronized to ring stage, starting with 100,000 parasites/μL (100,000 parasites; 10,000 parasites; 1000 parasites; 100 parasites and 10 parasites), run in duplicate.

### Analysis using PLS leave-one-out cross-validations (LOOCV)

The results from the qPCR were used to identify which individual mosquitoes had confirmed oocyst and sporozoite infections and their respective infection load. This information was then specified to each spectrum and these were randomly assigned to either a *training dataset* or a *test dataset* whilst ensuring the same proportion of different mosquito ages was found in each dataset. All uninfected mosquitoes were from the group that had been fed blood without viable gametocytes.

The *P. falciparum* detection model was trained and tested according to previously published methods [10] using partial least square (PLS) regression to develop a calibration based on a training data set, which was then used to predict the infection status of a separate set of samples contained in the test dataset, and therewith validate the prediction accuracy of the calibration. Leave-one-out cross validation (LOOCV) using partial least square regressions (PLS) were used to analyse the training dataset and to determine if NIR spectra of uninfected mosquitoes were distinct from *P. falciparum*-infected mosquitoes. LOOCV is a *k*-fold cross validation, with *k* equal to *n*, the number of spectra in a training dataset. That means that *n* separate times, the function approximator is trained on all the data except for one point and a prediction is made for that point. Multiple LOOCV with base on the training dataset were used to develop a calibration file which was then validated by testing its predictive accuracy on an independent test dataset.

**Fig. 1:**
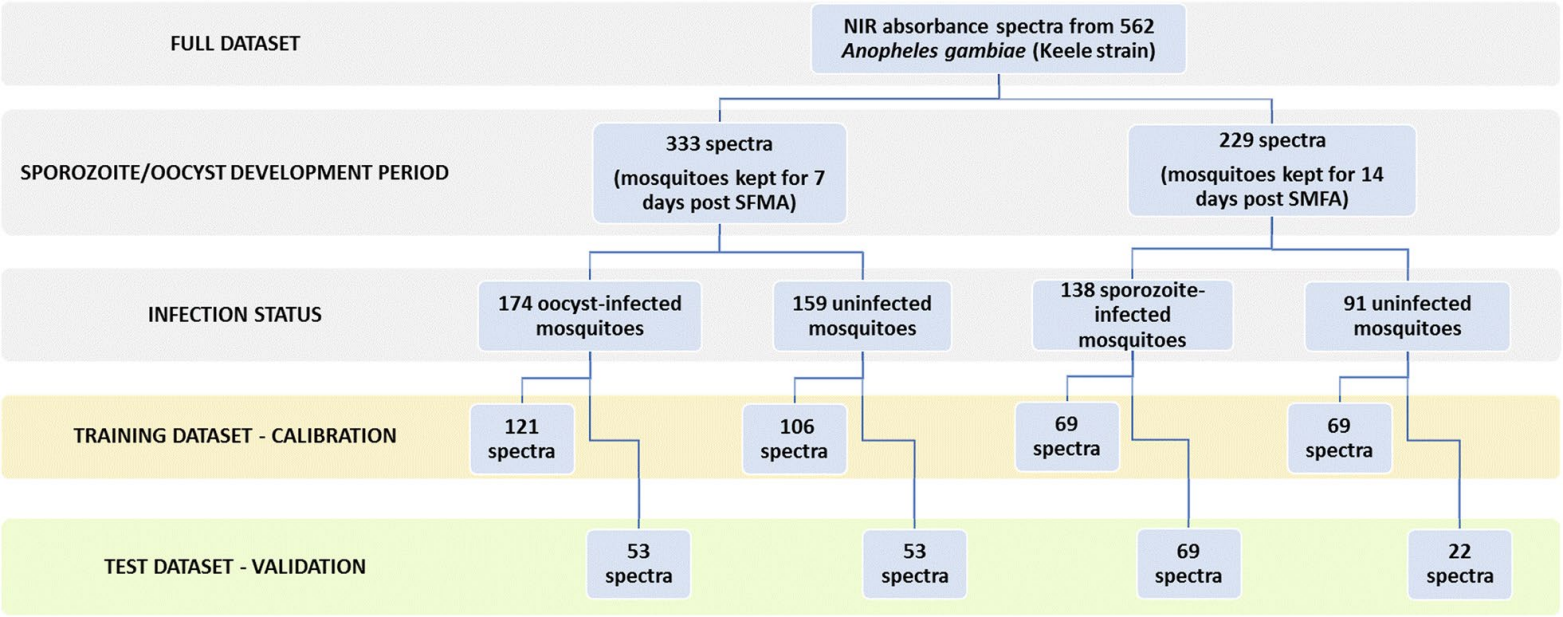
Study flow chart showing number of spectra collected, infection status and random assignment of spectra to either training or test dataset

Two separate LOOCV were run to investigate the prediction accuracy of oocyst-infected vs. uninfected, and sporozoite-infected and uninfected mosquitoes respectively. A total of 69 sporozoite-infected and 69 uninfected mosquitoes that had been kept for 14 days post SMFA were used to generate a calibration file. The same was done using spectra from 121 oocyst-infected mosquitoes and 110 uninfected mosquitoes kept for 7 days post SMFA. The models were run on Grams IQ software (Thermo Galactic, Salem, NH) and a total of 12 latent factors were selected by visualizing the prediction residual error sum of squares (PRESS) curve, and choosing the minimum number of factors needed to reduce the prediction error of the model without overfitting it. A latent factor is a standard term used to describe PLS models and does not directly translate as a peak or trough in NIR spectra. Latent variables are variables that are not directly observed but are rather inferred (through a mathematical model) from other variables that are observed (directly measured). A calibration file was generated on Grams IQ and loaded into IQPredict software to predict the infection status of the test dataset, composed of 69 sporozoite-infected and 22 uninfected, and, 53 oocyst-infected and 56 uninfected (Fig. 1). PLS scores were obtained based on the predicted probability of infection, with 1 = predicted as uninfected, 2 = predicted as infected and cut-off value of 1.5. Actual *vs* Predicted plots were drawn by plotting the actual constituent values (coded as 1 = uninfected and 2 = infected) on the x axis, and model predicted values on the y axis. Prediction values were generated according to previously published methods [10], values below 1.5 were considered to be predicted as uninfected and values equal to or above 1.5 predicted as infected.

### Analysis of prediction accuracy

Sensitivity was calculated to estimate of the model’s ability to detect the presence of infection and specificity as the model’s ability to detect the absence of infection. Accuracy was calculated as the overall prediction ability of the model (Table 1). Sensitivity, specificity, accuracy and respective exact Clopper-Pearson confidence intervals were calculated using MedCalc for Windows, version 18.0 (MedCalc Software, Ostend, Belgium). Cohen’s kappa was calculated in STATA/IC Version 13 as a measure of inter-rate agreement between qPCR (reference test) and NIRS.

**Table 1:** Sensitivity, specificity and accuracy as measures of the performance of a binary classification test

Sensitivity	Specificity	Accuracy
$\frac{TP}{TP + FN}$	$\frac{TN}{TN + FP}$	$\frac{TP + TN}{TP + TN + FP + FN}$

*TP* True positives, *TN* true negative, *FP* false positive, *FN* false negatives

The PLS scores of the predicted independent samples were analyzed using generalized linear mixed-effects model in STATA/IC Version 13.1. The response variable investigated was the PLS score generated from the PLS calibration models. The effects of infection presence and infection intensity (number of parasite genomes) on the PLS prediction value were investigated. Given that the age of a mosquito may affect NIRS spectra and therewith the PLS score, mosquito age was included as a random effect in the model. Regression coefficients for each factor, confidence intervals and p-values were reported. Model selection was done based on the Akaike information criterion (i.e. the lower the AIC value, the better the model).

## Results

### Experimental infections

Approximately 750 female *An. gambiae* (Keele line) [26] of different ages (3–6 days old) were offered a blood meal containing NF54 gametocyte cultures in standard membrane-feeding assays (SMFAs) in three independent replicate experiments. Control (uninfected) mosquitoes were generated by feeding approximately 450 mosquitoes the same blood after gametogenesis was completed. Both groups were represented with mosquitoes of similar ages, between 3 and 6 days old (see Additional file 1). Mosquitoes were maintained for 7 and 14 days under insectary conditions to allow oocyst (D7) and sporozoite (D14) development, on each day of sampling live mosquitoes were removed, killed and immediately scanned using NIRS.

Mosquitoes fed on infectious blood were analysed by quantitative polymerase chain reaction (qPCR) for intensity of infection. Additionally, 60 mosquitoes from the control groups (30 from feed 2 and 3, respectively) were also analysed by qPCR to confirm the absence of malaria infection. No mosquitoes from these control groups tested positive for infection.

The minimum number of parasite genomes detectable per mosquito was 10 parasite genomes/per μL of DNA extract, calculated from standard curves generated for each qPCR run using a 5-point tenfold serial dilution of DNA extracted from asexual 3D7 cultures synchronized to ring stage. This gave a threshold detection of ~ 500 parasite genomes per mosquito for the qPCR assay.

### Near infrared spectra selection

A total of 634 *An. gambiae* (Keele strain) were scanned using NIRS (Table 2). DNA was extracted and analyzed for *P. falciparum* infection by qPCR as described above. Samples with inconclusive qPCR results or poor spectra quality were excluded (n = 72). Poor quality or outlier spectra were visually identified by comparing them to all other spectra, and spectra that were prominently flat or prominently noisy were excluded, as described elsewhere [10]. Thus, NIR absorbance spectra and respective infection status data from a final total of 562 mosquitoes were used to estimate the accuracy of NIRS for prediction of malaria infection (Fig. 1).

**Table 2:** Description of the gametocytaemia used for each of the three standard membrane feeding assays (SMFA), number of days kept post blood feeding, number of mosquitoes processed by quantitative PCR (qPCR),  % prevalence, and the intensity of infection described as the median and interquartile range (IQR) of the number of parasite genomes per μl of DNA extract present in infected mosquitoes, excluding mosquitoes with no infection

SMFA	Estimated gametocytaemia	Day post- infectious blood meal	No. mosquitoes tested (n = 634)	Positive (N = 423)	% prevalence of infection	Intensity of infection: median number of parasite genomes and IQR.
1	1%	7	175	105	60.0%	680 (283–1625)
		14	n.d.	n.d.	n.d.	n.d.
2	0.1%	7	104	73	70.2%	456 (67–2052)
		14	99	47	47%	516 (211–6081)
3	0.1%	7	114	85	74.6%	2995 (3210–8881)
		14	142	113	80%	10,114 (2540–29,145)

### Model prediction accuracy

**Fig. 2:**
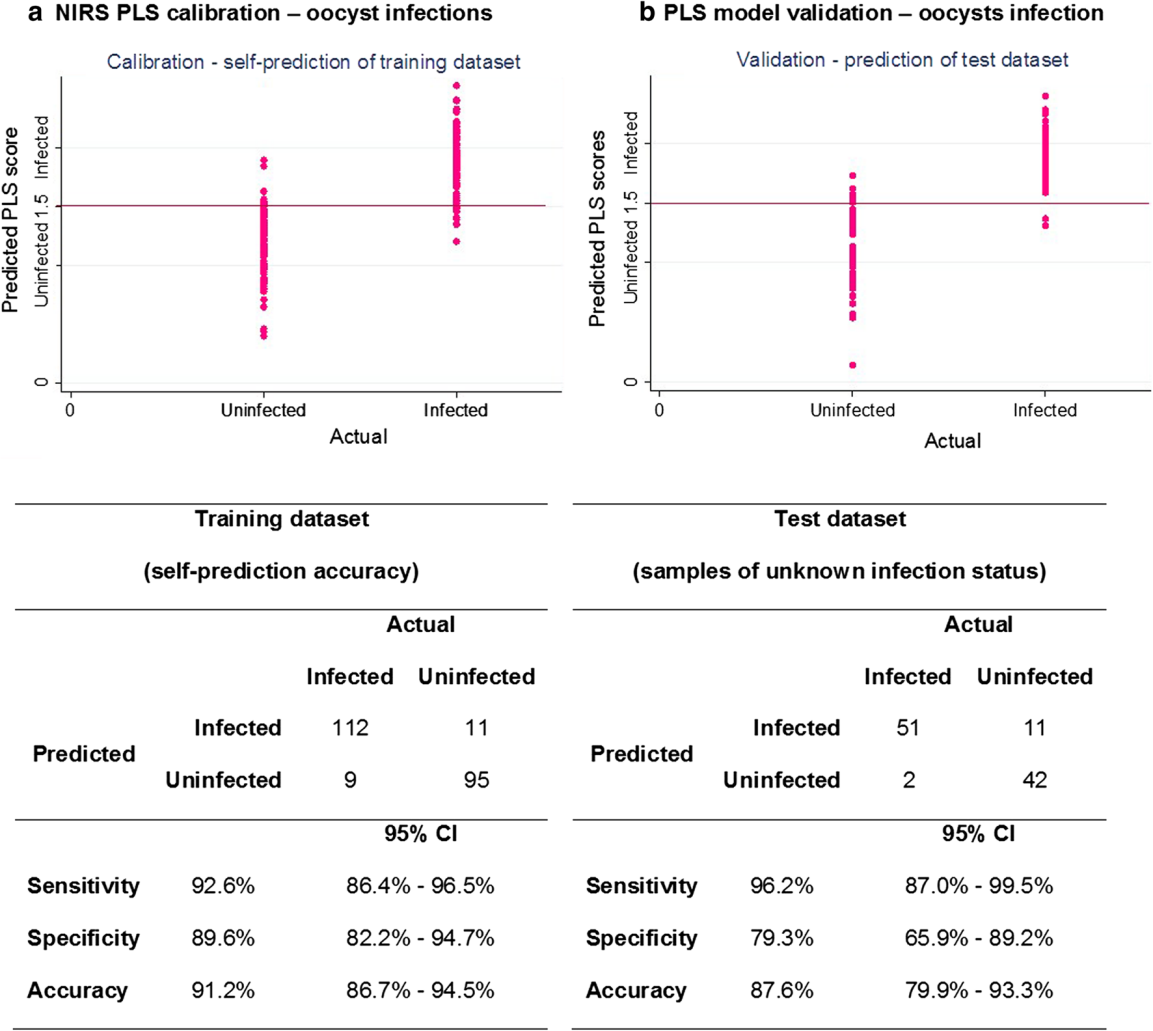
Actual versus predicted plots of oocyst infected mosquitoes investigating NIRS as diagnostic method. Sensitivity, specificity, accuracy and respective 95% confidence intervals of self-prediction of *P. falciparum*-infection in training dataset (**a**) and prediction of samples of unknown status in test dataset (**b**) (PLS scores: 1 = uninfected, 2 = infected and 1.5 as cut-off value)

**Fig. 3:**
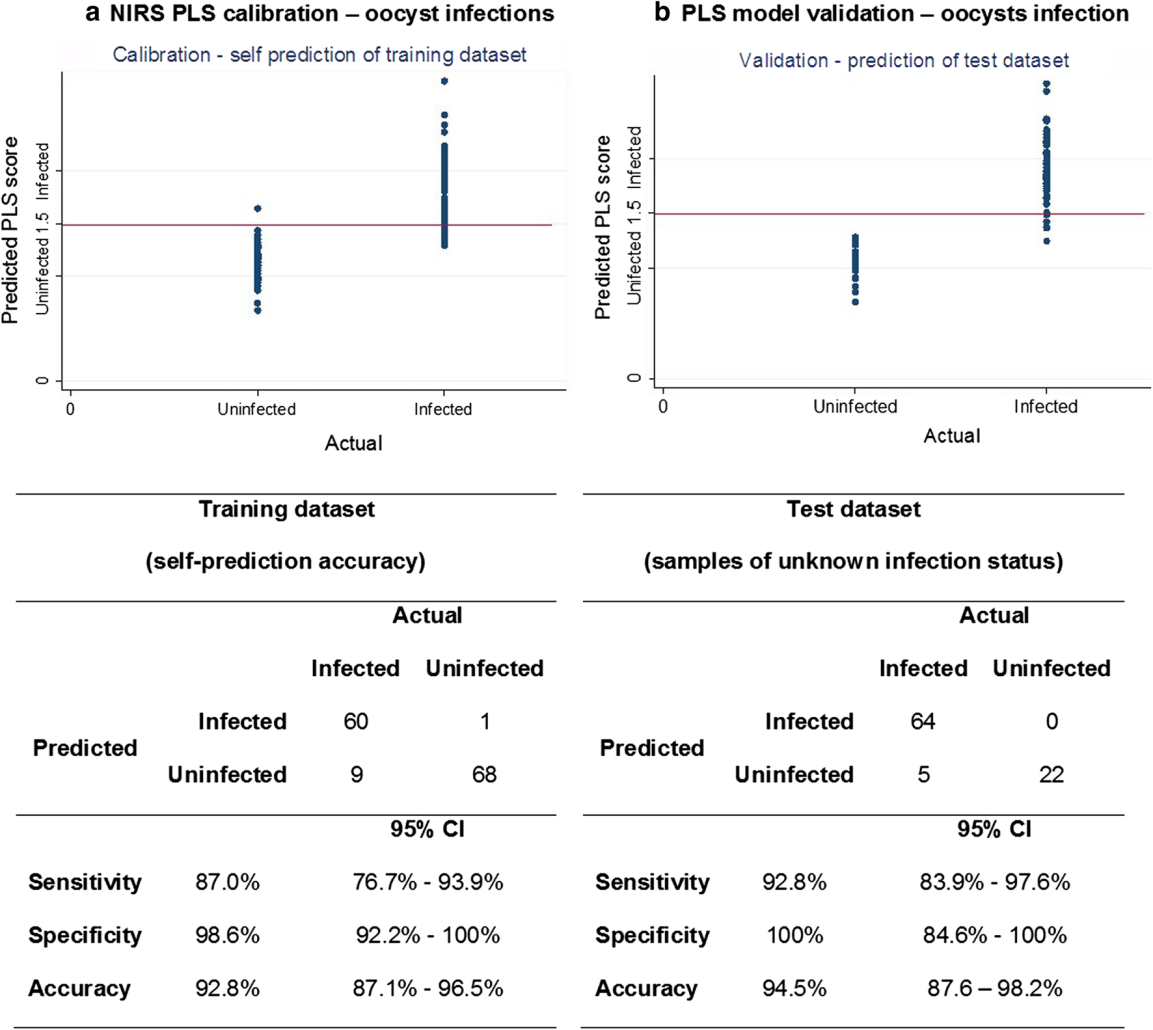
Actual versus predicted plots of sporozoite infected mosquitoes investigating NIRS as diagnostic method. Sensitivity, specificity, accuracy and respective 95% confidence intervals of self-prediction of *P. falciparum*-infection in training dataset (**a**) and prediction of samples of unknown status in test dataset (**b**) (PLS scores: 1 = uninfected, 2 = infected and 1.5 as cut-off value)

The relationship between spectra and infection was analyzed using partial least square regression (PLS). Training datasets were used to perform multiple leave-one-out cross validations (LOOCV) and develop two calibrations, one for prediction of oocyst infection and another for prediction of sporozoite infection. The calibrations were then validated using test datasets composed of samples with unknown infection status that had not been included in the calibration’s training dataset. The number of factors used in the calibration was 12, determined from the prediction residual error sum of squares (PRESS) and regression coefficient plots (see Additional file 1). In the PLS model, a value of “1” was assigned to all the actual uninfected samples whereas a value of “2” was assigned to the actual infected mosquitoes (infection as defined by the qPCR results). The PLS calibration derived components used to transform the original spectra of each predicted independent sample into a PLS score; a score value of 1.5 was considered as the threshold for correct or incorrect classification, meaning any mosquito with PLS score below 1.5 was predicted as uninfected and equal or greater than 1.5 was predicted as infected. The PLS model showed that NIR spectra from both oocyst and sporozoite-infected mosquitoes were distinct from their counterpart uninfected mosquitoes with 91.2% (86.7–94.5%) and 92.8% (87.1–96.5%) self-prediction accuracy respectively (Figs. 2a and 3a). When tested on samples with unknown infections status that had not been included in the training dataset, the calibration maintained high sensitivity and specificity at both detecting oocyst and sporozoite infection, with 87.7% (95% CI 79.9–93.3%; Cohen’s kappa = 0.75)) and 94.5% (95% CI 87.6–98.2%; Cohen’s kappa = 0.86) prediction accuracy respectively (Figs. 2b and 3b).

### Infection load and prediction accuracy

The parasite load in a mosquito is of epidemiological importance as there is evidence of a continual increase in transmission potential with increasing sporozoites numbers [30]. To test if the NIR prediction output scores were affected by parasite load, qPCR was used to estimate the relative number of parasite genomes in each infected mosquito (Table 3) and used to evaluate the calibration model’s accuracy.

**Table 3:** Generalized linear mixed-effects models investigating the effect of infection presence (infected or uninfected) and infection load (number of parasite genomes/μL of DNA extract quantified using qPCR) on the PLS score of the predicted samples including mosquito age as a random effect

	Coefficients	Robust standard error	z	95% Confidence intervals	P value
Oocyst infections					
Infection presence	0.67	0.13	5.11	0.41 to 0.93	< 0.001
Infection load	− 0.000003	− 0.000002	− 1.26	− 0.0000074 to 0.0000015	0.21
*Mosquito age* *(random effect)*	0.018	0.005	–	0.01 to 0.03	–
Sporozoite infections					
Infection presence	0.75	0.12	6.03	0.51 to 1.00	< 0.001
Infection load	0.0000019	0.0000003	5.82	0.0000013 to 0.0000025	< 0.001
*Mosquito age* *(random effect)*	0.007	0.008	–	0.0032 to 0.087	–

**Fig. 4:**
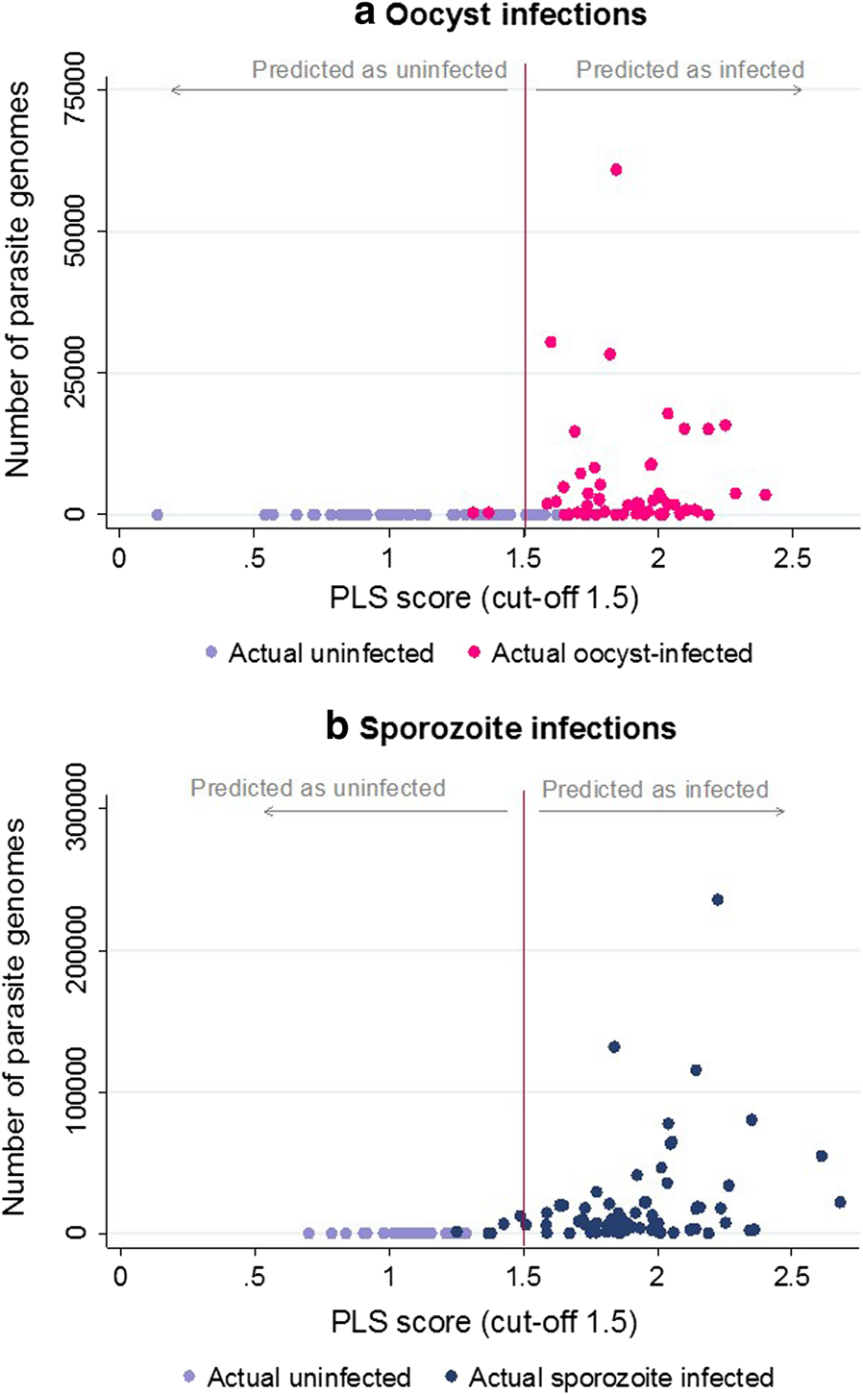
Intensity of *P. falciparum* oocyst (**a**) and sporozoite (**b**) infection, quantified as the number of parasite genomes per μl of DNA extract, in *A. gambiae* mosquitoes and prediction value score based on the predicted probability of infection, with 1 = predicted as not infected and 2 = predicted as infected (cut-off value of 1.5)

The oocyst-infected mosquitoes in the test data set had a range of infection loads (Median: 1925 parasite genomes/μL of DNA extract, IQR: [295 to 4883]). Two oocyst-infected mosquitoes were misclassified as uninfected, both of which had relatively low infection loads (357 and 389 parasite genomes/μL of DNA extract) (Fig. 4a). Generalized linear mixed-effects models were used to investigate the effect of infection load and infection presence on the PLS scores (response variable) of the predicted samples. The age of the mosquitoes on the day of the infectious feed was included as a random effect. It was observed that the presence of oocyst infection influenced the NIRS prediction score (Coefficient: 0.67; 95% CI 0.41 to 0.93; p < 0.001) but the infection load did not (Coefficient: − 0.000003; 95% CI − 0.0000074 to 0.0000015; *p* value: 0.21). The sporozoite-infected mosquitoes in the test dataset had a range of infection loads (Median: 8841 parasite genomes/μL of DNA extract, IQR: [2516 to 20,112]). Five sporozoite-infected mosquitoes were misclassified as uninfected: two presented with the lowest infection loads of the test dataset (33 and 38 parasite genomes/μL of DNA extract); the other three had relatively high infection loads (1156, 6660 and 12,591 parasite genomes/μL of DNA extract) (Fig. 4b). The presence of sporozoite significantly affected the PLS scores of the predicted samples (Coefficient: 0.75; 95% CI 0.51 to 1.00; p-value: < 0.001) as did the infection load (Coefficient: 0.0000019; 95% CI 0.0000013 to 0.0000025; p-value < 0.001).

## Discussion

This is the first study to show that NIRS can be used to accurately detect human malaria in *An. gambiae* mosquitoes. NIRS was able to predict oocyst infection with 87.7% accuracy (79.9–93.3%) and sporozoite infection with 94.5% accuracy (87.6–98.2%). The NIRS predictive accuracy for sporozoite infection of > 90% in this study concurs with previous work done using the rodent malaria in *An. stephensi*, which found that NIRS could detect the presence of sporozoites in infected mosquitoes with 77% accuracy [22]. Unlike the previous study, the present calibration model was also capable of identifying oocyst-infected mosquitoes. The PLS calibration of the present study was based on a narrower interval of the electromagnetic spectrum, 500 to 2400 nm, compared to 350 to 2500 nm previously used. This narrower range excludes noise present in the extremities of the spectra due to light source and sensor limitations and therewith improved the prediction accuracy of the calibration model. Furthermore, the previous study used spectra from mosquitoes that had been saturated with chloroform which was used to knock them down. This contamination led to clear chloroform peaks in the NIR spectra which may have added to the noise and reduced prediction accuracy of the calibration. Differences between the vector species and parasite species may also have played a role in the small discrepancy of predictive accuracy between studies. In addition, the experimental approach used in the present study, allowed to account for the potentially confounding effects of the infected blood meal, given that control group had been fed the same blood but with inactivated gametocytes.

Near infrared light is absorbed differently by diverse biochemical compounds which, in the mosquito, may consistently vary with between species, age and in this case infection status. It is hypothesized that biochemical changes occurring in the mosquito, as a consequence of *P. falciparum* infection, made it possible to distinguish between infected and uninfected mosquitoes using NIRS. Consistent differences between the NIR absorbance spectra of infected and uninfected mosquitoes may be related to the presence of parasite-specific molecules in the infected mosquitoes [31–33]. Also, it is possible that tissue changes may occur in the mosquitoes due to their immune response to the parasite which could have an effect on the biochemical composition of the mosquito [31]. Additionally, it is known that *Plasmodium* infection alters metabolic pathways in mosquitoes and leads to higher energy resource storage [34], which may lead to differences in NIRS spectra. More research is needed to better understand the underlying biochemical features that enable NIRS to distinguish between *Plasmodium*-infected and uninfected mosquitoes.

The prediction accuracy of the NIRS calibration to detect sporozoite infection was influenced not only by the presence of *P. falciparum* sporozoites but also the parasite load (number of parasite genomes). This was not the case of the calibration to detect oocysts, which was only significantly influenced by the presence of infection in the midgut. It is possible that slight differences in DNA extraction efficiency between samples may have affected the estimate number of parasite genomes in each insect sample and, therefore, it is imprudent to make conclusions on how strongly infection load may be influencing the PLS output scores. The performance accuracy of NIRS was similar to qPCR (sporozoite detection: Cohens kappa = 0.86; oocyst detection: Cohens’s kappa = 0.75). The strong inter-rate agreement between the two methods, suggests that NIRS may have similar sensitivity and specificity to qPCR at detecting malaria sporozoites in the mosquito host. ELISA is less specific than PCR [35], however due to its low-cost and ease, it is routinely the assay chosen by surveillance programs to measure the proportion of mosquitoes that carry sporozoites and the entomological inoculation rate (EIR). It is possible that EIR estimates could be improved by using a more accurate diagnostic test. ELISA commonly uses pooled samples to reduce costs and time, given that infection rates are usually below 2%. In case of an ELISA well positive for infection, it is assumed that it arises from one mosquito in the respective pool. In contrast, NIRS could be used on all samples since sample processing is less-costly and faster; it takes approximately 20 s to position and collect NIR spectra from one mosquito, allowing around 100 mosquitoes to be analyzed in 30 min. In addition, the method is completely non-destructive which permits using the sample for further tests if needed. However, a direct comparison of NIRS and ELISA was not the objective of this study, as the latter method does not allow quantification of parasite infection which was needed to evaluate if NIRS prediction was affected by *P. falciparum* infection load. Presently NIRS still requires further optimization and validation in the field before being considered as a possible replacement for ELISA in surveillance programmes. Furthermore, the experiment described used fresh mosquitoes. Analyzing mosquitoes directly after sorting and morphological identification may be feasible for research programmes, but less so for control or surveillance programmes, which would benefit from evaluating different preservation methods.

It is noteworthy that the NIRS instrument is a rugged piece of equipment, which does not require special installation or frequent maintenance, and does not necessarily need to be installed in a laboratory. It was originally designed to be carried to the field to collect plant and soil spectra, can be transported in a Pelican case (55 × 42 × 32 cm), and requires only a power supply, which can be supplied by battery packs that are included with the instrument or from a 12 V vehicle power outlet. It can be assembled or packed in minutes, and if used frequently, left on the bench simply protected with a vinyl cover as is done with a compound microscope. Generating calibrations requires expert knowledge and technical skills, however, if a calibration file is readily available, predicting a sample’s classification group is simple, requiring only brief training. The current field-deployable NIR spectrometer costs around 55,000 USD but given that analysis requires no consumables or reagents and is high-throughput, the investment could be quickly paid off, particularly given the potential of the same technology for age-grading and species identification.

While the results presented in this paper are promising, NIRS calibrations generated using lab-reared mosquitoes do not necessarily represent the diversity of vectors in the field, providing no guarantee of the robustness of the method when tested on wild-caught mosquitoes. Calibrations must be based on training datasets that capture the diversity of field-mosquitoes reducing confounders that may affect the classification accuracy, including, different mosquito species, age, infection, size, insecticide resistance status, microbiome, and origin. Scale-up will require the assembly of training datasets to generate calibrations that capture this variability, by including a comprehensive range of mosquitoes characterized by diverse geographical, ecological, and epidemiological backgrounds. This approach is likely to narrow the factors needed for prediction by explaining sources of noise and variability in the model that are not directly related to infection and therewith increase prediction accuracy. Depending on how well the signal caused by infection presence is conserved, this could lead to location-, country- or region-specific calibrations for infection detection.

## Conclusions

NIRS is a promising technology that may provide an accurate and high-throughput solution to monitoring malaria transmission in the vector as progression towards elimination is made. Such a tool may revolutionize how entomological data is used by research programmes given that the same test can report various entomological parameters, including age, species and infection status, therewith compiling vast amounts of information of epidemiological importance to increase understanding of how vector populations and malaria transmission are changing. Future research efforts and resources need to be directed at evaluating the best way of generating and optimizing calibrations based on wild-caught mosquitoes for each entomological parameter, and validating these using specimens from different ecological and geographical regions.

## Additional file

**Additional file 1.** GRAMS output figures and table describing the mosquitoes contained in the training and test datasets by age (on the SMFA day) and infection load (number of parasite genomes).
