# Correction to: Detection of *Plasmodium falciparum* infected *Anopheles gambiae* using near-infrared spectroscopy

**DOI:** 10.1186/s12936-019-2776-0

**Published:** 2019-04-17

**Authors:** Marta F. Maia, Melissa Kapulu, Michelle Muthui, Martin G. Wagah, Heather M. Ferguson, Floyd E. Dowell, Francesco Baldini, Lisa Ranford-Cartwright

**Affiliations:** 1 Swiss Tropical and Public Health Institute, Socinstrasse 57, 4020 Basel, Switzerland; 2 University of Basel, Petersplatz 1, 4001 Basel, Switzerland; 3 KEMRI Wellcome Trust Research Programme, P.O. Box 230, Kilifi, 80108 Kenya; 4 Centre for Tropical Medicine and Global Health, Nuffield Department of Medicine, University of Oxford, Old Road Campus Roosevelt Drive, Oxford, OX3 7FZ UK; 5 Department of Public Health, School of Human and Health Sciences, Pwani University, Kilifi, Kenya; 6 Institute of Biodiversity, Animal Health and Comparative Medicine, University of Glasgow, Graham Kerr Building, Glasgow, G12 8QQ UK; 7 USDA, Agricultural Research Service, Center for Grain and Animal Health Research, 1515 College Avenue, Manhattan, KS 66502 USA

## Correction to: Malar J (2019) 18:85 10.1186/s12936-019-2719-9

Following publication of the original article [1], it was flagged that the name of the author Lisa Ranford-Cartwright had been (incorrectly) given as ‘Lisa-Ranford Cartwright. That is, the hyphen had been incorrectly positioned.

The original article has been updated to correct this error. 
